# Global Mycotoxin Occurrence in Feed: A Ten-Year Survey

**DOI:** 10.3390/toxins11070375

**Published:** 2019-06-27

**Authors:** Christiane Gruber-Dorninger, Timothy Jenkins, Gerd Schatzmayr

**Affiliations:** BIOMIN Research Center, Technopark 1, 3430 Tulln, Austria

**Keywords:** mycotoxin, animal, feed, maize, weather, climate, Europe, Asia, Africa, America

## Abstract

Mycotoxins contaminating animal feed can exert toxic effects in animals and be transferred into animal products. Therefore, mycotoxin occurrence in feed should be monitored. To this end, we performed a large-scale global survey of mycotoxin contamination in feed and assessed regional differences and year-to-year variation of mycotoxin occurrence. Concentrations of aflatoxin B_1_, zearalenone, fumonisins, ochratoxin A, deoxynivalenol, and T-2 toxin were analyzed in 74,821 samples of feed and feed raw materials (e.g., maize, wheat, soybean) collected from 100 countries from 2008 to 2017. In total, 88% of the samples were contaminated with at least one mycotoxin. Mycotoxin occurrence showed distinct regional trends and climate was a key determinant governing these trends. In most regions, the majority of samples complied with maximum levels and guidance values for mycotoxins in animal feed that are in effect in the European Union. However, 41.1%, 38.5%, and 20.9% of samples from South Asia, Sub-Saharan Africa, and Southeast Asia, respectively, exceeded the maximum level for aflatoxin B_1_ (20 µg/kg). In several regions, mycotoxin concentrations in maize showed a pronounced year-to-year variation that could be explained by rainfall or temperature during sensitive periods of grain development. A large fraction of samples (64%) was co-contaminated with ≥ 2 mycotoxins. Most frequently observed mycotoxin mixtures were combinations of deoxynivalenol, zearalenone, and fumonisins, as well as fumonisins and aflatoxin B_1_. Deoxynivalenol and zearalenone concentrations were correlated in maize and wheat. In conclusion, according to an extensive global survey, mycotoxin (co-)contamination of animal feed is common, shows regional trends, and is governed in part by climate and weather.

## 1. Introduction

Mycotoxins are toxic fungal secondary metabolites frequently found as contaminants of food and feed. Mycotoxigenic fungi infest crop plants in the field or agricultural commodities during storage. The most common mycotoxins are aflatoxins (e.g., aflatoxin B_1_; AFB_1_), fumonisins, zearalenone (ZEN), type B trichothecenes (e.g., deoxynivalenol; DON), type A trichothecenes (e.g., T-2 toxin; T-2), and ochratoxin A (OTA). These mycotoxins are known to exert toxic effects in farm animals, causing distress and reduced productivity [1]. Furthermore, some mycotoxins may carryover in livestock products, such as meat, eggs, and milk [2], thereby compromising the safety of human consumers. To prevent negative effects on animals and consumers, many countries regulate mycotoxin concentrations in feed. In the European Union (EU), for example, maximum levels are enforced for AFB_1_ [3] and guidance values have been stipulated for fumonisins, ZEN, DON, and OTA [4]. Mycotoxin concentrations in feed should be continuously monitored to support risk assessment.

Multiple factors determine the contamination of agricultural commodities with mycotoxins. Mycotoxin occurrence varies between crops, as fungal species and strains differ in their ability to infest a particular host, and it varies between varieties of the same plant species, as varieties show different levels of susceptibility or resistance to fungal infestation. Furthermore, environmental conditions, such as temperature and humidity, affect the infestation of crop plants with mycotoxigenic fungi and mycotoxin production by these fungi and, therefore, climate and weather are strong determinants of mycotoxin contamination [5]. Moreover, agricultural practices, timing of harvest, and post-harvest handling of crops affect mycotoxin formation [6].

Crops may be infested with multiple strains of mycotoxigenic fungi and most fungal strains produce more than one type of mycotoxin. Therefore, co-contamination of agricultural commodities with multiple mycotoxins is frequently observed [7]. When feed raw materials are mixed to produce compound feed, mycotoxin co-contamination becomes even more likely. If mycotoxins co-occur, their combined toxic effect may be additive, synergistic, or antagonistic, i.e., equal to, greater than, or lower than the summed effects of the individual mycotoxins [8,9]. Scientific interest in biological effects of mycotoxin mixtures has been increasing in recent years, but knowledge on this topic is still scarce. Monitoring mycotoxin co-occurrence enables identifying the most prevalent mycotoxin mixtures and, thus, can help to prioritize research efforts.

Since 2004, BIOMIN has been conducting a global survey program to monitor mycotoxin contamination of animal feed. Studies on these data have been published previously [10,11,12,13,14]. In the study presented here, we analyzed global occurrence and co-occurrence of AFB_1_, fumonisins, ZEN DON, OTA, and T-2 in 74,821 samples of finished feed and feed raw materials such as maize, wheat, barley, and soybean collected from 100 countries during a 10-year period. We compared mycotoxin occurrence in 15 geographic regions covering most of the globe and analyzed the year-to-year variation of mycotoxin concentrations in finished feed and maize from each region. To investigate the effect of weather on mycotoxin occurrence, we compared historical weather data from maize growing areas to year-to-year variation of mycotoxin concentrations in maize. This is, to the best of our knowledge, the largest dataset of mycotoxin concentrations in feed and the most comprehensive assessment of regional trends of mycotoxin occurrence published to date.

## 2. Results

### 2.1. Global Mycotoxin Occurrence

In total, 74,821 samples collected from 100 countries were analyzed for AFB_1_, fumonisins, ZEN DON, OTA, and T-2. Of the samples tested for ≥ 3 mycotoxins, 88% were contaminated with at least one mycotoxin. The *Fusarium* mycotoxins DON, fumonisins, and ZEN were most prevalent and were detected in 64%, 60%, and 45% of all samples, respectively (Table 1). AFB_1_, T-2, and OTA were detected in 23%, 19%, and 15% of the samples, respectively (Table 1). Fumonisins and DON showed the highest median concentrations, namely 723 µg/kg and 388 µg/kg, respectively (Table 1).

We compared global mycotoxin occurrence in different commodities, including finished feed, maize, maize dried distillers grains with solubles (DDGS), maize silage, soybean grains, soybean meal, wheat, barley, and rice. Mycotoxin occurrence differed between these commodities. Finished feed was among the commodities showing the highest percentage of positive samples for every mycotoxin analyzed (Table 1). In maize, fumonisins showed a higher prevalence (80% positive samples) and higher median value (1300 µg/kg) than in any other commodity (Table 1). Maize furthermore showed a high prevalence of DON (67% positive samples), ZEN (44% positive samples), and AFB_1_ (24% positive samples). Similar to maize, maize DDGS showed a high prevalence of fumonisins (78% positive samples), DON (83% positive samples), and ZEN (75% positive samples). Median concentrations of DON (1490 µg/kg) and AFB_1_ (11 µg/kg) were higher in maize DDGS than in maize or any other commodity analyzed in this survey (Table 1). Furthermore, the prevalence of OTA (22% positive samples) was higher in maize DDGS than in most of the other commodities (Table 1). As in maize, fumonisins, ZEN, and DON were the most frequently detected mycotoxins in maize silage. However, the prevalence and median concentration of fumonisins were markedly lower in maize silage than in maize (Table 1). In both soybean grains and soybean meal, ZEN was the most prevalent mycotoxin, detected in 36% and 61% of samples, respectively (Table 1). Furthermore, DON, AFB_1_, and T-2 were detected in 29%, 20%, and 18% of soybean grain samples and in 31%, 29%, and 33% of soybean meal samples, respectively. In wheat and barley, DON (65% and 61%, respectively), T-2 (22% and 22%, respectively), and ZEN (33% and 20%, respectively) were the most frequently detected mycotoxins (Table 1). Rice showed a higher percentage of samples contaminated with AFB_1_ (31%) than any other commodity (Table 1). Furthermore, 34% and 27% of rice samples were contaminated with ZEN and DON, respectively.

### 2.2. Regional Mycotoxin Occurrence

**Figure 1:**
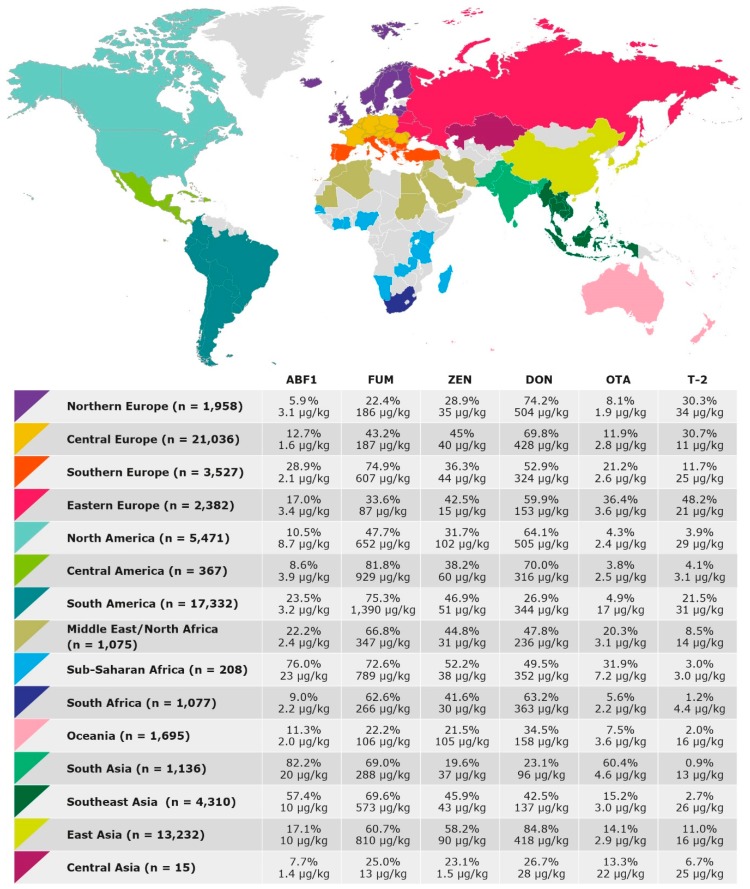
Occurrence of mycotoxins in 15 geographic regions. For each region, countries of sample origin are labeled in the map using a distinct color. The legend indicates percentage of positive samples and median of positive samples for each mycotoxin in each region. Each row represents one region and is labeled using the distinct color corresponding to the respective region in the map. n–sample number; AFB_1_–aflatoxin B_1_; DON–deoxynivalenol; ZEN–zearalenone; FUM–fumonisins (sum of fumonisins B_1_, B_2_,and B_3_); OTA–ochratoxin A; T-2–T-2 toxin.

To elucidate regional trends of mycotoxin occurrence we broke down the global dataset into datasets of 15 geographic regions (i.e., Northern Europe, Central Europe, Southern Europe, Eastern Europe, North America, Central America, South America, Middle East/North Africa, Sub-Saharan Africa, South Africa, Oceania, South Asia, East Asia, Southeast Asia, and Central Asia). For each of these regions, prevalence and median concentrations of AFB_1_, fumonisins, ZEN, DON, OTA, and T-2 are shown in Figure 1. For risk assessment, the percentages of samples exceeding EU regulatory limits or guidance values for mycotoxins in feed are shown in Table 2. Many countries enforce legal limits for mycotoxins in feed that differ from the EU limits. However, to allow a comparison between regions, samples from all regions were compared to EU limits in this study.

**Figure 2:**
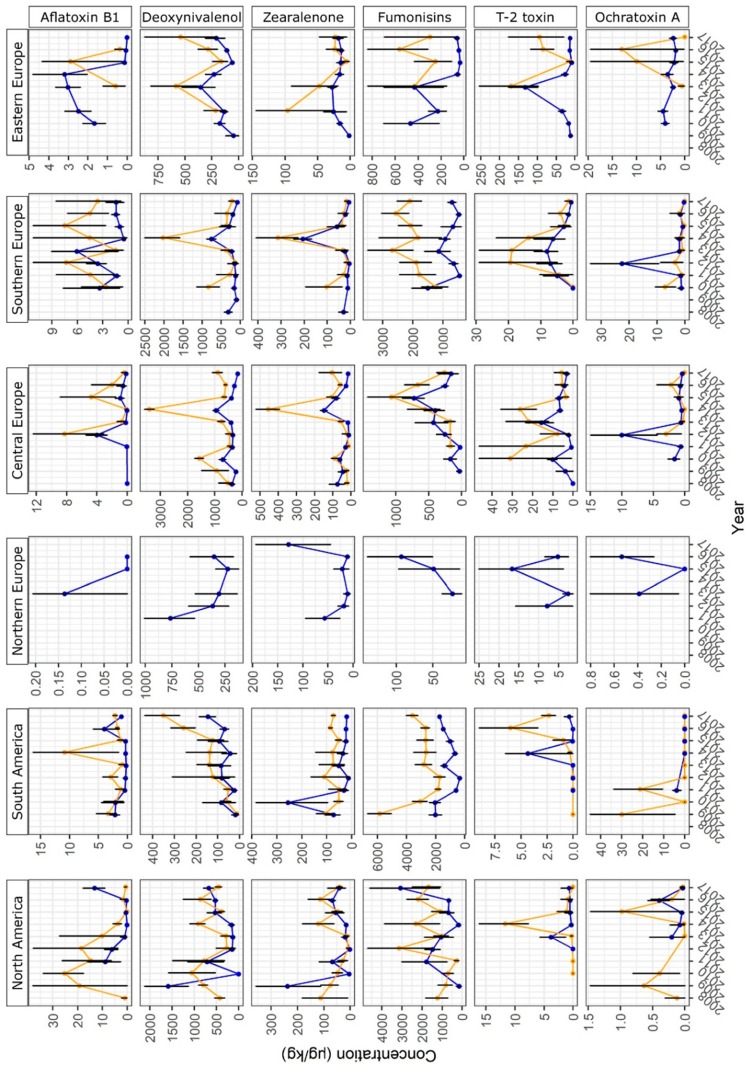
Year-to-year variation of mycotoxin concentrations in North America, South America, Northern Europe, Central Europe, Southern Europe, and Eastern Europe. The vertical axis shows mean concentrations of mycotoxins (Bayesian mean with error bars for 95% confidence level; see the Materials and Methods section for details on statistical analysis). The horizontal axis shows harvest years 2008–2017. Taking into account approximate seasons of crop growth and harvest, a year was defined to start in April and end in March of the subsequent calendar year for South America or to start in October and end in September of the subsequent calendar year for all other regions. Yellow circles and lines indicate maize samples. Blue circles and lines indicate finished feed samples. Data points are shown if ≥ 20 samples per year were available.

**Figure 3:**
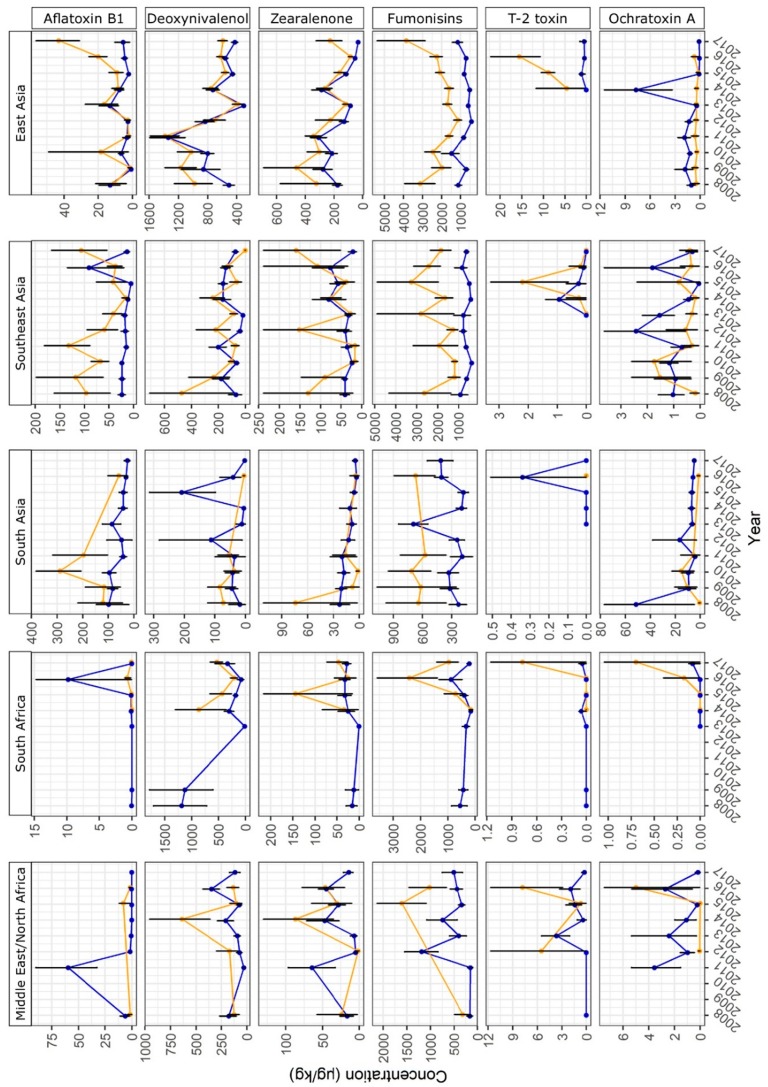
Year-to-year variation of mycotoxin concentrations in Middle East/North Africa, South Africa, South Asia, Southeast Asia, and East Asia. The vertical axis shows mean concentrations of mycotoxins (Bayesian mean with error bars for 95% confidence level; see Materials and Methods section for details on statistical analysis). The horizontal axis shows harvest years 2008–2017. Taking into account approximate seasons of crop growth and harvest, a year was defined to start in April and end in March of the subsequent calendar year for South Africa or to start in October and end in September of the subsequent calendar year for all other regions. Yellow circles and lines indicate maize samples. Blue circles and lines indicate finished feed samples. Data points are shown if ≥ 20 samples per year were available.

The year-to-year variation of mycotoxin concentrations in maize and finished feed samples collected from different regions is shown in Figure 2 and Figure 3. For Central America, Sub-Saharan Africa, Central Asia, and Oceania, year-to-year variation of mycotoxin concentrations in maize and finished feed was not investigated, as lower sample numbers did not allow this analysis. The results for each region are described in detail in the following sections.

#### 2.2.1. Northern Europe

Trichothecenes were prevalent in samples collected from Northern Europe (Figure 1). DON was detected in 74.2% of the samples and T-2 was detected in 30.3% of the samples. T-2 showed a median concentration of 34 µg/kg, which was the highest median concentration obtained for any region. Furthermore, a relatively high median concentration of 504 µg/kg was detected for DON and 21.5% of samples did not comply with the lowest EU guidance value for DON, stipulated for the most sensitive animal species (Table 2). Just 1.0% of the samples did not comply with the highest EU guidance value for DON, stipulated for the most tolerant animal species.

#### 2.2.2. Central Europe

In Central European samples, trichothecenes were prevalent (Figure 1). In total, 69.8% and 30.7% of the samples were found to be contaminated with DON and T-2, respectively. DON reached a relatively high median level of 428 µg/kg. Furthermore, ZEN and fumonisins were detected in 45.0% and 43.2% of the samples, respectively. The lowest EU guidance values for DON and ZEN were exceeded by 20.4% and 13.0% of the samples, respectively (Table 2). Just 0.9% and 0.4% of the samples did not comply with the highest EU guidance values for DON and ZEN, respectively. In maize, mean concentrations of DON and ZEN were significantly higher in 2014 than in the other years (Figure 2).

#### 2.2.3. Southern Europe

Fumonisins were the most prevalent mycotoxins in samples from Southern Europe. They were detected in 74.9% of samples at a median concentration of 607 µg/kg. Furthermore, DON was detected in 52.9% of the samples and ZEN was detected in 36.3% of the samples. For DON and ZEN, 11.7% and 11.8% of the samples exceeded the lowest EU guidance value, with 0.5% and 0.2%, exceeding the highest EU guidance value, respectively. As in Central Europe, mean concentrations of DON and ZEN in maize peaked in 2014 (Figure 2). AFB_1_ was more prevalent in Southern Europe than in the other European regions (28.9% compared to 5.9–17.0% positive samples, Figure 1). Furthermore, the fractions of samples exceeding lowest and highest EU regulatory limits for AFB_1_ were higher in this region than in the rest of Europe (Table 2). The highest regulatory limit was exceeded in 2.1% of cases.

#### 2.2.4. Eastern Europe

Trichothecenes were prevalent in samples from Eastern Europe (Figure 1). DON was detected in 59.9% of the samples and T-2 was detected in 48.2% of the samples and the latter was therefore more prevalent in this dataset than in datasets from any other region. Furthermore, ZEN was detected in 42.5% of the samples and OTA showed a relatively high prevalence of 36.4%.

#### 2.2.5. North America

DON, fumonisins, and ZEN were the most prevalent mycotoxins in samples from North America, detected in 64.1%, 47.7%, and 31.7% of the samples, respectively. Compared to other regions (Figure 1), DON and ZEN showed relatively high median concentrations of 505 µg/kg and 102 µg/kg, and 19.1% and 16.8% of the samples exceeded the lowest EU guidance value, but only 0.8% and 0.6% of samples exceeded the highest EU guidance values, respectively (Table 2).

#### 2.2.6. Central America

In samples from Central America, fumonisins were more prevalent than in samples from any other region (Figure 1). They were detected in 81.8% of samples at a relatively high median concentration of 929 µg/kg. Furthermore, DON was prevalent being detected in 70.0% of the samples and ZEN was detected in 38.2% of the samples.

#### 2.2.7. South America

In the South American dataset, fumonisins were detected in a high fraction of samples (75.3%) at a median concentration of 1390 µg/kg. This was the highest median concentration obtained for fumonisins in any region (Figure 1). Furthermore, 8.4% of the samples exceeded the lowest EU guidance value for fumonisins, with 0.2% of the samples exceeding the highest EU guidance value (Table 2). Fumonisin concentrations in maize were particularly high in 2009 and tended to increase between 2012 and 2017 (Figure 2). ZEN was detected in 46.9% of samples. T-2 was detected in 21.5% of samples at a relatively high median concentration of 31 µg/kg.

#### 2.2.8. Middle East/North Africa

In samples from Middle East and North Africa, fumonisins, DON, and ZEN were the most frequently detected mycotoxins with 66.8%, 47.8%, and 44.8% positive samples, respectively (Figure 1). AFB_1_ concentrations in finished feed were significantly higher in 2011 than in the other years (Figure 3). However, it has to be noted that all high values obtained in 2011 were from a group of samples from Mauritius. Therefore, the high average that year is of doubtful significance for the wider region. Furthermore, the finished feed samples may have contained imported ingredients or have been affected by storage conditions and consequently, the detected AFB_1_ concentrations may not be representative of local crops.

#### 2.2.9. Sub-Saharan Africa

AFB_1_ was detected in 76.0% of samples from Sub-Saharan Africa at a median concentration of 23 µg/kg, the highest median concentration detected in any region (Figure 1). Consequently, 59.1% of these samples exceeded the lowest EU regulatory limit for AFB_1_ in feed and still 38.5% of the samples did not comply with the highest EU regulatory limit of 20 µg/kg (Table 2). *Fusarium* mycotoxins fumonisins, ZEN, and DON were prevalent in this region as well, and detected in 72.6%, 52.2%, and 49.5% of the samples, respectively. 

#### 2.2.10. South Africa

*Fusarium* mycotoxins DON, fumonisins, and ZEN were the most prevalent mycotoxins in South African samples and detected in 63.2%, 62.6%, and 41.6% of samples, respectively (Figure 1). Fumonisin concentrations in maize were high and DON concentrations were low in samples from 2016 (Figure 3). This has been reported and discussed in a recent publication on a dataset of South African feed samples derived from the BIOMIN Mycotoxin Survey that overlaps with the dataset presented here [13].

#### 2.2.11. Oceania

In samples from Oceania, DON was the most frequently detected mycotoxin, with 34.5% of positive samples (Figure 1). ZEN was detected in a comparatively low fraction of samples (21.5%), but reached a high median concentration of 105 µg/kg. Accordingly, 11.1% of samples exceeded the lowest EU guidance value for ZEN in feed (Table 2). Most samples (99.3%) complied with the highest EU guidance value.

#### 2.2.12. South Asia

AFB_1_ was detected in 82.2% of samples from South Asia, which was the highest percentage of positive samples found in any region (Figure 1). Furthermore, AFB_1_ reached a high median concentration of 20 µg/kg. Accordingly, high fractions of samples, i.e., 61.1% and 41.1%, did not comply with the lowest and highest EU regulatory limits for AFB_1_ in feed, respectively (Table 2). OTA was detected in 60.4% of the samples, which was again the highest percentage of positive samples detected in any dataset. However, nearly all samples (99.6%) complied with the most stringent EU guidance value for OTA in feed. In addition to AFB_1_ and OTA, fumonisins were prevalent in South Asia, being detected in 69% of the samples.

#### 2.2.13. Southeast Asia

AFB_1_ was prevalent in samples from Southeast Asia. It was detected in 57.4% of the samples at a median concentration of 10 µg/kg and 37.9% and 20.9% of the samples did not comply with the lowest and highest EU regulatory limits for AFB_1_, respectively (Table 2). AFB_1_ concentrations in maize were particularly high in 2008–2011 and in 2017 (Figure 3). Apart from AFB_1_, *Fusarium* mycotoxins were prevalent in the dataset from Southeast Asia. Fumonisins, ZEN, and DON were detected in 69.6%, 45.9%, and 42.5% of the samples, respectively.

#### 2.2.14. East Asia

In samples from East Asia, DON and ZEN were more prevalent (84.8% and 58.2% positive samples, respectively) than in samples from any other region (Figure 1). Relatively high median concentrations of 418 µg/kg and 90 µg/kg were detected for DON and ZEN, respectively. In total, 20.6% and 27.3% of samples exceeded the lowest EU guidance value and 0.7% and 1.3% of samples exceeded the highest EU guidance value for DON and ZEN, respectively (Table 2). DON levels were low in 2014 relative to other years in this region (Figure 3). In addition to DON and ZEN, fumonisins were prevalent in the East Asian dataset with 60.7% of positive samples and a relatively high median concentration of 810 µg/kg (Figure 1). Fumonisin concentrations peaked in samples from 2017 (Figure 3). AFB_1_ was detected in a lower fraction of samples (17.1%) than in South Asia and Southeast Asia, but at a relatively high median concentration of 10 µg/kg. Accordingly, 10.2% and 6.6% of the samples exceeded the lowest and highest EU regulatory limit for AFB_1_ in feed, respectively. The mean concentration of AFB_1_ was higher in 2017 than in previous years (Figure 3).

#### 2.2.15. Central Asia

For Central Asia, we only had a small dataset of 15 samples available. These samples did not show notable trends for prevalence or median concentrations of mycotoxins (Figure 1). The limited dataset did not allow more detailed analyses as performed for the other regions.

### 2.3. Co-Occurrence of Mycotoxins

In total, 64% of all samples tested for ≥ 3 mycotoxins were found to contain ≥ 2 mycotoxins. To analyze the co-occurrence of mycotoxins in different commodities, we calculated the fraction of samples contaminated with either combination of two mycotoxins for finished feed, maize, and wheat. In case of finished feed, combinations of DON, fumonisins, and ZEN were most frequently observed (Table 3). DON and ZEN, DON and fumonisins, and ZEN and fumonisins co-occurred in 48%, 48%, and 43% of the samples, respectively. In maize, the same mycotoxin combinations were most prevalent (Table 3). Co-occurrence of DON and ZEN, DON and fumonisins, and ZEN and fumonisins was detected in 39%, 49%, and 37% of the samples, respectively. Furthermore, AFB_1_ and fumonisins co-occurred in 22% and 24% of finished feed and maize samples, respectively. In wheat, DON and ZEN was the most frequently observed combination, detected in 28% of the samples (Table 3).

**Figure 4:**
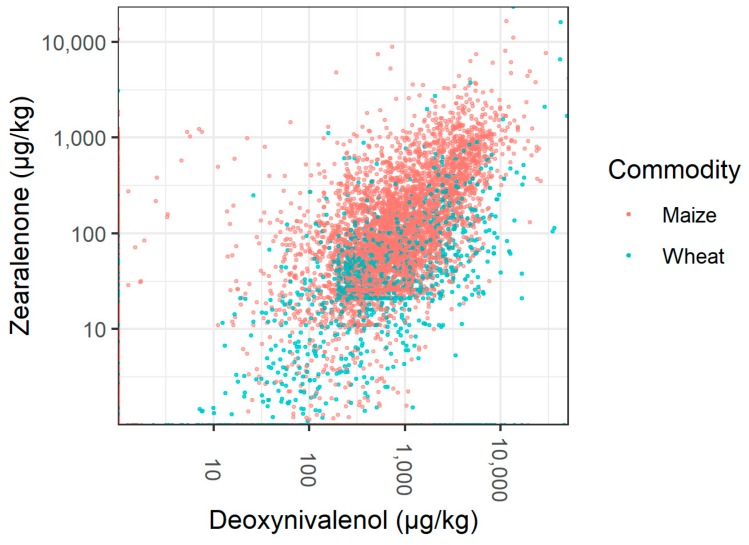
Correlation of zearalenone and deoxynivalenol concentrations in samples of maize (red circles) and wheat (turquoise circles). Both axes are in logarithmic scale.

We calculated the correlation of mycotoxin concentrations for any combination of two mycotoxins in maize and wheat. Concentrations of DON and ZEN showed a positive correlation with a correlation coefficient (on log-transformed data) of 0.483 and 0.375 in maize and wheat, respectively (Figure 4). All other combinations showed correlation coefficients of ≤ 0.2.

To investigate regional trends of mycotoxin co-occurrence, we calculated for each region defined in Figure 1 the fraction of samples contaminated with either combination of two mycotoxins for finished feed, maize, and wheat. As in the global dataset, dual combinations of DON, ZEN, and fumonisins were the most frequently detected mycotoxin combinations in these commodities in most regions (data not shown). However, AFB_1_ and fumonisins was the most frequently detected mycotoxin combination in finished feed from Sub-Saharan Africa (89% positive samples) and Southeast Asia (62% positive samples) and in maize from Sub-Saharan Africa (60% positive samples), Southeast Asia (62% positive samples), South Asia (64% positive samples), and Oceania (29% positive samples). Furthermore, AFB_1_ and OTA was the most frequently detected combination in finished feed from South Asia (81% positive samples).

## 3. Discussion

### 3.1. Global Patterns of Mycotoxin Occurrence in Different Commodities

Each feed raw material showed a distinct pattern of mycotoxin occurrence (Table 1). Maize showed a particularly high prevalence and high levels of fumonisins and frequently contained DON, ZEN, and AFB_1_. Wheat and barley were mainly contaminated with DON and additionally contained T-2 and ZEN. Soybean and soybean meal were mainly contaminated with ZEN, DON, T-2, and AFB_1_. In rice, ZEN, AFB_1_, and DON were most frequently detected. These patterns reflect well-known associations of certain fungal species with these crop plants. For example, fumonisin producer *F. verticillioides* is a known pathogen of maize [15] and DON and ZEN producers *F. culmorum* and *F. graminearum* infest assorted cereal species, including maize, wheat, barley, and rice [16].

Compared to the raw materials, finished feed showed a high percentage of positive samples for all mycotoxins (Table 1). This is not surprising, since finished feed is a blend of different commodities and therefore can be expected to contain a blend of mycotoxins occurring in these commodities. For example, maize and maize products are commonly added to finished feed as main ingredients. Consequently, both maize and finished feed showed a high prevalence of fumonisins in our survey, whereas this was not observed for other feed raw materials (Table 1).

Maize DDGS showed the highest median levels of DON and AFB_1_ of all commodities (Table 1). DDGS are a by-product of bioethanol production. Mycotoxins present in the starting material are enriched in DDGS [17]. For example, the DON concentration by dry weight has been reported to be three times higher in DDGS than in the initial grain [18,19]. Although the global datasets of maize and maize DDGS samples analyzed in this study are not directly comparable, as they contain samples from varying geographical regions over a 10-year period, and consequently the maize samples analyzed may not resemble the maize used as starting material for DDGS production, our results confirm higher mycotoxin concentrations in maize DDGS compared to maize grains. The notable exception in our data is for fumonisins, which are known in the literature to concentrate in DDGS at levels around three times the original grain levels [20]. The opposite pattern in our results is likely to be largely related to high sample numbers of corn grain and lack of DDGS from high-fumonisin regions of South America (see Materials and Methods).

### 3.2. Effects of Climate and Weather on Regional Patterns of Mycotoxin Occurrence

Prevalence and median concentrations of mycotoxins varied between regions (Figure 1). Several factors may contribute to these differences. As discussed above, susceptibility to mycotoxin contamination varies between crops and as datasets from different regions contained different proportions of samples from each commodity (Table 4) reflecting crops preferentially grown or consumed in each region, mycotoxin occurrence may vary accordingly. Furthermore, pre- and post-harvest agricultural practices that affect fungal growth and mycotoxin production may vary between regions. Importantly, regions may show different trends of mycotoxin occurrence due to differences in climatic conditions affecting mycotoxin formation during crop plant development and during the storage of crops.

**Figure 5:**
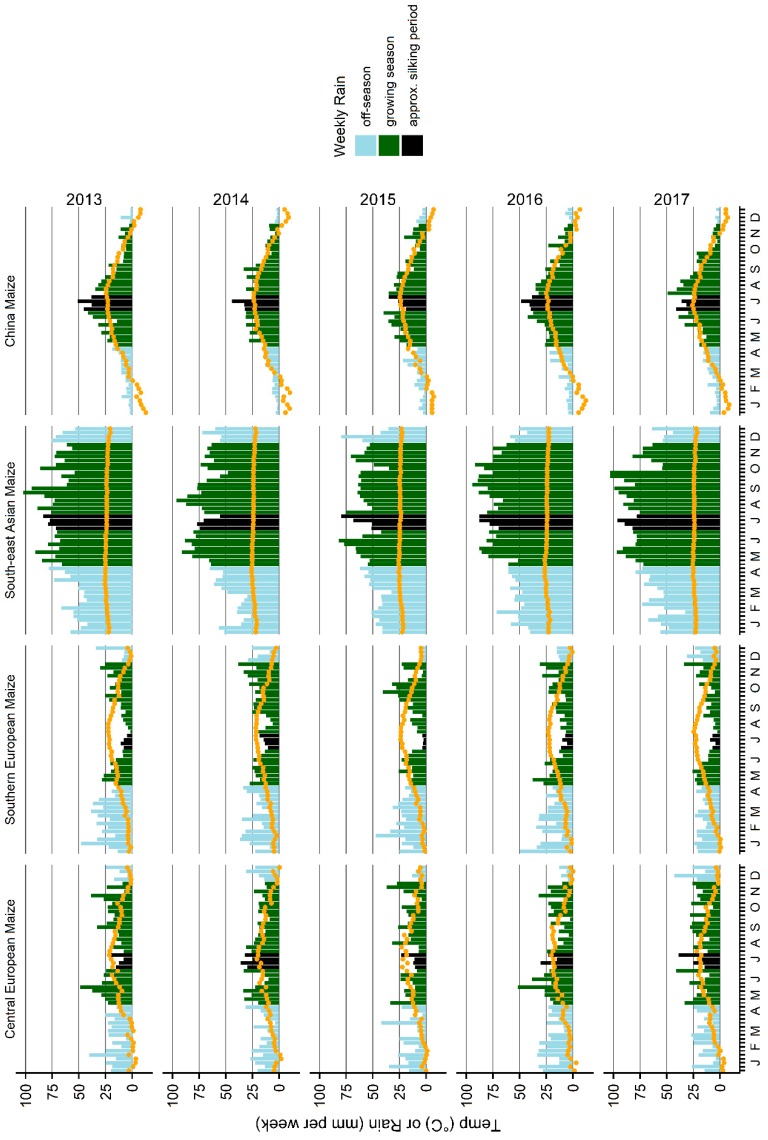
Rainfall and temperature in maize-growing areas of Central Europe, Southern Europe, Southeast Asia, and China in 2013–2017. Bars indicate weekly total rainfall. Green, black, and light blue bars correspond to the approximate maize growing season, silking period, and off-season, respectively (these timings can differ between regions and management practice, and are affected by weather). Orange dots indicate weekly mean temperature. The vertical axis shows rainfall (in mm) and temperature in (°C). The horizontal axis indicates the months (J–January, F–February, M–March, A–April, M–May, J–June, J–July, A–August, S–September, O–October, N–November, D–December).

As climatic conditions are the main determinants of mycotoxin formation in crops, we discuss the impact of climate on regional occurrence of AFB_1_, DON, ZEN, and fumonisins in more detail in the following sections. Furthermore, we discuss the effect of weather on the year-to-year variation of mycotoxin concentrations in maize. To this end, we compare trends of mycotoxin concentrations in maize from Southeast Asia, Central Europe, Southern Europe, and East Asia (Figure 3 and Figure 4) to rainfall and temperature measured in maize growing areas in these regions in 2013–2017 (Figure 5).

#### 3.2.1. Aflatoxin B_1_

In samples from Sub-Saharan Africa, Southeast Asia, and South Asia, AFB_1_ was prevalent and detected at high concentrations (Figure 1) often exceeding the highest EU regulatory limit for AFB_1_ in feed (Table 2). These data indicate that AFB_1_ is a significant burden for animal production in these regions. Climatic conditions in these regions (mainly tropical or sub-tropical) facilitate aflatoxin contamination of crops. On the one hand, infestation of crop plants with aflatoxigenic *Aspergillus* spp. and the production of aflatoxins in the growing plant is favored by drought stress, i.e., periods of high temperature and low humidity [21]. On the other hand, exposure to high temperatures and high moisture leading up to harvest [5] and during storage of crops [22] facilitates fungal growth and aflatoxin production. Either scenario is common in tropical and subtropical climates.

It can be expected that many of the AFB_1_-positive samples collected from Europe were imported from other parts of the world, as climatic conditions in temperate Europe generally do not favor infestation of crop plants with aflatoxigenic fungi. However, AFB_1_ contamination of maize grown in Southern European countries has been reported in recent years [23,24,25,26]. Hot and dry conditions necessary for *Aspergillus flavus* infestation of maize mainly prevail in Europe below 45° North latitude [27] and therefore in the region defined in this study as Southern Europe (Figure 1). Accordingly, in this study, prevalence of AFB_1_ was higher in samples from Southern Europe than in samples from the other European regions (Figure 1) and 2.1% of samples exceeded the highest EU maximum level for AFB_1_ in feed (Table 2). AFB_1_ contamination of crops in Southern Europe should continue to be monitored closely, as occasional high levels occur and this may increase in the future due to climate change [27,28].

AFB_1_ concentrations in maize varied from year to year in several regions (Figure 2 and Figure 3) and some of this variation could be traced back to a variation in weather conditions. In Southeast Asia, AFB_1_ concentrations were significantly higher in maize harvested in 2017 than in maize harvested in 2013–2016 (Figure 3). These higher AFB_1_ levels may reflect the relatively high rainfall in August and September 2017 leading up to harvest (Figure 5; weeks 31–39: 783.1 mm in 2017; 551.5–720.4 mm in 2013–2016). In maize from East Asia, AFB_1_ concentrations were significantly higher in 2017 than in previous years. This may be due to a higher temperature during the approximate silking period of maize (July) in the core Chinese maize growing areas in 2017 compared to 2013–2016 (Figure 5; mean temperature in weeks 27–30: 24.6 °C in 2017; 23.1–23.7 °C in 2013–2016). These two examples illustrate the effect of hot and humid weather conditions on AFB_1_ contamination levels in maize.

#### 3.2.2. Deoxynivalenol and Zearalenone

Rainfall and mild temperatures during the flowering and maturation periods were shown to favor infestation of wheat and maize with *F. graminearum* and *F. culmorum* and DON contamination [5,29]. Accordingly, higher DON concentrations were detected in samples from the temperate regions North America, Northern Europe, Central Europe, and East Asia (Figure 1).

For several regions analyzed in this study, the year-to-year variation of concentrations of DON and ZEN (also produced by *F. graminearum* and *F. culmorum*) in maize could be correlated with rainfall. DON and ZEN concentrations were exceptionally high in maize harvested in 2014 in Central Europe (Figure 2). These peaks in DON and ZEN concentrations corresponded with higher than usual rainfall in July 2014 (Figure 5; weeks 27–30: 122.7 mm in 2014; 56.4–94.9 mm in 2013, 2015, 2016, 2017), i.e., during the main silking period of maize, ongoing relatively high rainfall in August (Figure 5; weeks 31–35: 122.0 mm in 2014; 62.6–84.6 mm in 2013, 2015, 2016, 2017), and moderate rainfall in September (Figure 5). High rainfall during the silking period could have facilitated infestation of maize plants with *Fusarium* spp., whereas ongoing rainfall in the lead-up to harvest would have meant an extended period of suitable grain moisture levels for continued fungal growth and mycotoxin production within the grain. Same as in Central Europe, DON and ZEN showed relatively high levels in Southern European maize harvested in 2014 (Figure 2), which also coincided with heavier than usual rainfall in July (Figure 5; weeks 27–30: 56.0 mm in 2014; 11.8–33.3 mm in 2013, 2015, 2016, 2017) and August (Figure 5; weeks 31–35: 60.6 mm in 2014; 29.8–47.4 mm in 2013, 2015, 2016, 2017). In East Asian maize, DON and ZEN levels were relatively low in maize harvested in 2013 (Figure 3), which may reflect the relatively lower levels of rainfall in August and September (leading up to harvest) that year in the core Chinese maize growing areas compared to the following years (Figure 5; weeks 31–39: 210.3 mm in 2013; 215.1–270.6 mm in 2014–2017). Overall, these observations confirm a key impact of rainfall on DON and ZEN contamination levels in maize.

#### 3.2.3. Fumonisins

Infestation of maize with *F. verticillioides* and consequent fumonisin contamination is facilitated by high temperatures and low precipitation around silking [30,31,32,33]. It is therefore not surprising that regions with a hot climate such as South America, Central America or Sub-Saharan Africa showed particularly high levels of contamination in this survey (Figure 1). Furthermore, in the case of Europe, highest median concentrations were detected in Southern Europe (Figure 1), the warmest and driest region of the continent.

Year-to-year trends of fumonisin concentrations in maize could be correlated with patterns observed in weather data. In Central Europe, fumonisin concentrations peaked in maize harvested in 2015 (Figure 2). This could be related to warmer temperatures in July (during silking) and August (in the lead-up to harvest) that year than observed in other years (Figure 5; mean temperature in weeks 27–30: 19.8 °C in 2015; 18.2–19.2 °C in 2013, 2014, 2016, 2017; mean temperature in weeks 31–35: 19.8 °C in 2015; 16.7–18.7 °C in 2013, 2014, 2016, 2017). Furthermore, the amount of rainfall in July (weeks 27–30) was lower in 2015 (56.4 mm) than in the other years (58.7–122.7 mm). A peak in fumonisin concentration observed in East Asian maize harvested in 2017 could be associated with relatively high temperatures in the core Chinese maize growing areas in July of that year (Figure 5; mean temperature in weeks 27–30: 24.6 °C in 2017; 23.1–23.7 °C in 2013–2016). In this case, the amount of rainfall was average compared to 2013–2016 (Figure 5; weeks 27–30: 134.3 mm in 2017; 102.3–172.7 mm in 2013–2016). In summary, fumonisin concentration peaks in Central European and East Asian maize could be related to high temperatures during the silking period.

### 3.3. Co-Occurrence of Mycotoxins

Mycotoxin co-occurrence was frequently observed with ≥ 2 mycotoxins detected in 64% of all samples tested for ≥ 3 mycotoxins. Risk assessment and regulation usually target single mycotoxins, not mycotoxin mixtures. However, the results of this study indicate that mycotoxin co-contamination of feed and consequently, mycotoxin co-exposure of animals, is the rule rather than the exception. Therefore, it is important to consider the combined toxic effects of mycotoxins.

Most frequently observed mycotoxin combinations in finished feed, maize, and wheat were combinations of *Fusarium* mycotoxins DON, ZEN, and fumonisins (Table 3). Furthermore, DON and ZEN concentrations showed a positive correlation in maize and wheat (Figure 4). As DON and ZEN are both produced by the same fungal species, i.e., *F. graminearum* and *F. culmorum*, a correlation of their concentrations in agricultural commodities is not surprising. In published studies investigating the combined effect of DON and ZEN in animals, additive, synergistic, and antagonistic effects have been observed. The type of interaction may vary with the investigated parameter, the animal species, age, sex, or nutritional status of the animals, administered mycotoxin dose, as well as duration and route of mycotoxin administration [8]. Additive or synergistic effects of DON and ZEN were reported for parameters of immune function in mice and pigs [34,35,36], parameters of liver health and antioxidant function in mice and rats [37,38], and parameters of oxidative stress in the spleen [36], brain [39], and kidneys [40] of mice. Antagonistic effects were reported for parameters of immune function in pigs [35] and parameters of liver health [37] and liver metabolism [41] in mice. Mixtures of fumonisins and DON or fumonisins and ZEN have also been shown to exert different types of combined effects in animals or in vitro, including additive and synergistic effects [8,9].

AFB_1_ and fumonisins frequently co-occurred in maize and finished feed from Sub-Saharan Africa, Southeast Asia, and South Asia. As for the mycotoxin combinations discussed above, any type of combined effect has been reported for AFB_1_ and fumonisins in animals. Importantly, according to the assessment by Grenier and Oswald [8], a synergistic negative effect on zootechnical parameters (e.g., body weight gain, feed conversion, egg weight) has been observed in several animal species including pigs [42,43], chickens [44,45], quail [46], and rabbits [47]. Furthermore, AFB_1_ and fumonisins have been shown to induce liver lesions in an additive or synergistic manner (e.g., [47,48,49]), and, when administered sequentially, fumonisins promoted liver cancer initiated by AFB_1_ in rats [50,51] and trout [52].

In summary, published studies on the effects of mycotoxin combinations detected frequently in this survey suggest a stronger toxic effect of the mixtures compared to each individual mycotoxin. Mycotoxin dosage and mode of administration varied between studies and, in many cases, the mycotoxin challenge applied may not be comparable to dietary exposure to mycotoxin concentrations reported here. The high prevalence of mixtures containing DON, ZEN, and fumonisins or AFB_1_ and fumonisins in feed necessitates further investigation of combined effects of these mycotoxins in animals, especially for dietary exposure to concentrations commonly detected in feed. Such studies would be important to clarify if there is a need for regulation of mycotoxin mixtures in animal feed.

## 4. Conclusions

In conclusion, analysis of 74,821 samples collected from 100 countries indicated that mycotoxins are almost ubiquitously present in feed. Each feed raw material showed a distinct pattern of mycotoxin contamination according to well-known associations of certain fungal pathogens with certain plant hosts. As a blend of raw materials, finished feed showed a comparatively high prevalence of all mycotoxins. 

Governed by climate as one key determinant, each region showed a distinct mycotoxin occurrence pattern and, therefore, faces its own challenges with respect to mycotoxin contamination of feed. Mycotoxin concentrations mostly complied with EU regulatory limits and guidance values stipulated for the most resistant animal species. However, as a notable exception, large fractions of samples from Sub-Saharan Africa, Southeast Asia, and South Asia were contaminated with high AFB_1_ concentrations exceeding the EU maximum level for the most resistant species, indicating a threat for animal and human health.

Mycotoxin contamination levels in maize from each region varied from year to year and weather conditions (i.e., rainfall and temperature) during sensitive periods of flowering and grain development were found to explain some of this variation. Our data suggest that extreme weather conditions during these periods may cause mycotoxin contamination levels far in excess of concentrations typically observed in a given region, as exemplified by a sudden increase in DON and ZEN concentrations in Central European and Southern European maize in 2014 that coincided with high rainfall in July and August of that year.

Results of this survey indicate that co-occurrence of mycotoxins is the rule rather than the exception. Consequently, the toxicological effect of frequently detected mycotoxin mixtures (most importantly combinations of DON, ZEN, and fumonisins, as well as, in some regions, the combination of fumonisins and AFB_1_) should be investigated more closely, especially with respect to dietary exposure to concentrations commonly detected in feed. 

## 5. Materials and Methods

### 5.1. Collection of Feed Samples

In total, 74,821 feed samples were collected from 100 countries from January 2008–December 2017. The countries were classified into 15 regions: Northern Europe (Denmark, Finland, Iceland, Ireland, Latvia, Lithuania, Norway, Sweden, United Kingdom); Central Europe (Austria, Belgium, Czech Republic, France, Germany, Hungary, Moldova, Poland, Romania, Slovakia, Slovenia, Switzerland, The Netherlands); Southern Europe (Bosnia and Herzegovina, Bulgaria, Croatia, Cyprus, Greece, Italy, Portugal, Serbia, Spain, Turkey); Eastern Europe (Belarus, Russia, Ukraine); North America (Canada, USA); Central America (Costa Rica, Cuba, Dominican Republic, Guatemala, Honduras, Mexico, Nicaragua, Panama); South America (Argentina, Bolivia, Brazil, Chile, Colombia, Ecuador, Paraguay, Peru, Uruguay); Middle East/North Africa (Algeria, Egypt, Iran, Israel, Jordan, Kuwait, Lebanon, Mauritania, Morocco, Saudi Arabia, Sudan, Syria, Tunisia, UAE, Yemen); Sub-Saharan Africa (Ghana, Ivory Coast, Kenya, Madagascar, Namibia, Nigeria, Senegal, Tanzania, Uganda, Zambia); South Africa (South Africa); Oceania (Australia, New Zealand); South Asia (Bangladesh, India, Nepal, Pakistan, Sri Lanka); Southeast Asia (Indonesia, Laos, Malaysia, Myanmar, Philippines, Singapore, Thailand, Vietnam); East Asia (China, Japan, Korea, Taiwan); and Central Asia (Kazakhstan). Sample numbers per commodity and region are given in Table 4.

Sampling, milling of samples, and homogenization of samples was performed as described previously [14]. Paper bags or bags with ventilation were used as sample containers to avoid humidity build-up. Samples that showed a high moisture content were dried. Samples were immediately sent to the laboratory for analysis.

### 5.2. Mycotoxin Analysis

Mycotoxin concentrations were analyzed using the methods specified in Table 5.

For ZEN, DON, fumonisin B_1_, fumonisin B_2_, fumonisin B_3_, OTA, and T-2, the threshold of relevant concentration was defined as either > 1.0 µg/kg or > limit of detection, whichever was higher. For AFB_1_, the threshold of relevant concentration was defined as either > 0.5 µg/kg or > limit of detection, whichever was higher. Correlations between mycotoxin concentrations were analyzed using ggpairs in the ggally package [53] in R software, version 3.3.0 [54]. For this analysis, results below the limit of detection were treated as zero values. Timeline graphs were constructed using ggplot2 package [55] and data was summarized with the dplyr package [56].

### 5.3. Analysis of Weather Data

Gridded weather data from 2013, 2014, 2015, 2016, and 2017 was accessed from the Cleaned Observations dataset of The Weather Company (IBM) calculated by The Weather Company algorithms and summarized as weekly total rainfall and weekly mean temperature for the world regions. Weather data for four of the regions was visually analyzed for patterns coincident with annual changes in mycotoxin concentrations. The weather data is displayed in Figure 5.

Central European weather data included 51 grid locations from Austria, 21 from Belgium, 50 from Czech Republic, 328 from France, 250 from Germany, 49 from Hungary, one from Luxemburg, 31 from the Netherlands, 223 from Poland, 135 from Romania, 32 from Slovakia, 11 from Slovenia, and 23 from Switzerland.

Southern European gridded weather locations included 16 from Albania, 28 from Bosnia and Herzegovina, 57 from Bulgaria, 37 from Croatia, eight from Cyprus, 107 from Greece, 228 from Italy, four from Kosovo, 12 from Macedonia, one from Malta, one from Monaco, seven from Montenegro, 50 from Portugal, 38 from Serbia, 263 from Spain, and 366 from Turkey.

Southeast Asian weather data was based on two gridded locations from Brunei, 51 from Cambodia, one from Christmas Island, one from Cocos (Keeling) Islands, six from East Timor, 773 from Indonesia, 68 from Laos, 105 from Malaysia, 234 from Myanmar (Burma), two from Palau, 180 from Papua New Guinea, 172 from the Philippines, 154 from Thailand, and 108 from Vietnam.

East Asian weather data was based on the mainland Chinese maize growing districts with 50 grid locations from Anhui, six from Beijing, 163 from Gansu, 78 from Guangxi, 58 from Guizhou, 17 from Hainan, 87 from Hebei, 268 from Heilongjiang, 65 from Henan, 66 from Hubei, 71 from Hunan, 597 from Inner Mongolia, 50 from Jiangsu, 99 from Jilin, 76 from Liaoning, 21 from Ningsia Hui Autonomous Region, 80 from Shaanxi, 67 from Shandong, 65 from Shanxi, 203 from Sichuan, five from Tianjin, 766 from Xinjiang, and 121 from Yunnan.

## Figures and Tables

**Table 1:** Global mycotoxin occurrence in different commodities.

Mycotoxin	n^1^	Positive Samples^2^		Median of Positives (µg/kg)	1^st^ Quartile of Positives (µg/kg)	3^rd^ Quartile of Positives (µg/kg)	Maximum (µg/kg)
		n^1^	%				
*All samples*							
Aflatoxin B_1_	51,475	11,941	23	4	2	17	10,918
Fumonisins^3^	46,477	27,890	60	723	240	1858	290,517
Zearalenone	61,413	27,559	45	55	25	147	105,000
Deoxynivalenol	59,107	37,940	64	388	200	885	84,860
Ochratoxin A	32,271	4858	15	3	2	7	2000
T-2 Toxin	27,850	5289	19	22	8	40	3,051
*Finished feed*							
Aflatoxin B_1_	16,563	4251	26	6	2	23	10,918
Fumonisins^3^	16,285	11,825	73	555	198	1297	290,517
Zearalenone	19,171	10,676	56	41	20	102	9432
Deoxynivalenol	18,649	13,004	70	294	134	600	32,893
Ochratoxin A	11,990	2801	23	3	2	6	1582
T-2 Toxin	9884	2246	23	10	4	22	1300
*Maize*							
Aflatoxin B_1_	15,889	3835	24	4	1	22	6,105
Fumonisins^3^	12,965	10,397	80	1300	520	2,940	218,883
Zearalenone	15,860	7002	44	77	33	217	16,495
Deoxynivalenol	12,660	8486	67	520	260	1240	51,374
Ochratoxin A	6388	334	5	3	2	14	889
T-2 Toxin	6087	727	12	25	11	53	978
*Maize DDGS*							
Aflatoxin B_1_	320	62	19	11	4	20	340
Fumonisins^3^	329	256	78	814	398	1870	26,828
Zearalenone	368	275	75	102	60	237	2896
Deoxynivalenol	381	316	83	1490	574	2579	84,860
Ochratoxin A	280	62	22	4	2	11	53
T-2 Toxin	52	3	6	40	35	43	46
*Maize silage*							
Aflatoxin B_1_	3104	188	6	2	1	4	342
Fumonisins^3^	3010	1114	37	138	45	416	7090
Zearalenone	3735	1508	40	84	34	201	6239
Deoxynivalenol	4206	2588	62	474	219	1092	34,861
Ochratoxin A	2830	161	6	3	2	6	69
T-2 Toxin	1800	58	3	20	10	51	685
*Soybean grains*							
Aflatoxin B_1_	916	186	20	1	1	2	74
Fumonisins^3^	794	135	17	68	29	223	7023
Zearalenone	1024	364	36	43	26	71	4336
Deoxynivalenol	975	284	29	416	160	640	5500
Ochratoxin A	718	86	12	3	2	7	46
T-2 Toxin	557	102	18	29	23	37	317
*Soybean meal*							
Aflatoxin B_1_	1692	490	29	2	1	4	109
Fumonisins^3^	1475	336	23	104	31	290	7210
Zearalenone	1767	1072	61	47	33	83	3720
Deoxynivalenol	802	247	31	119	25	424	5600
Ochratoxin A	606	82	14	4	2	10	141
T-2 Toxin	975	324	33	33	25	44	754
*Wheat*							
Aflatoxin B_1_	2210	221	10	1	1	3	161
Fumonisins^3^	2219	304	14	117	31	246	28,278
Zearalenone	4925	1624	33	34	20	75	23,278
Deoxynivalenol	5949	3866	65	369	218	865	49,307
Ochratoxin A	1973	172	9	3	2	5	364
T-2 Toxin	1993	439	22	25	13	35	1300
*Barley*							
Aflatoxin B_1_	727	64	9	1	1	2	120
Fumonisins^3^	776	65	8	53	17	366	10,485
Zearalenone	3129	637	20	25	20	58	8952
Deoxynivalenol	4046	2468	61	359	234	750	35,000
Ochratoxin A	730	46	6	3	2	9	150
T-2 Toxin	1225	272	22	26	9	51	404
*Rice*							
Aflatoxin B_1_	205	63	31	5	2	14	113
Fumonisins^3^	244	49	20	142	63	382	6895
Zearalenone	220	74	34	60	34	107	1530
Deoxynivalenol	226	60	27	266	87	436	3859
Ochratoxin A	230	32	14	3	2	5	20
T-2 Toxin	54	5	9	9	8	26	30

^1^ Sample number; ^2^ Positive samples are defined as > limit of detection, excluding aflatoxins below 0.5 ng/g and other mycotoxins below 1 ng/g; ^3^ Sum of fumonisins B_1_, B_2_, and B_3_.

**Table 2:** Percentage of samples exceeding lowest and highest maximum levels or guidance values for mycotoxins in feed that are in effect in the European Union.

Region	Aflatoxin B_1_^1^		Fumonisins^1^		Zearalenone^1^		Deoxynivalenol^1^		Ochratoxin A^1^	
	% Exceeding		% Exceeding		% Exceeding		% Exceeding		% Exceeding	
	5 µg/kg	20 µg/kg	5000 µg/kg	60,000 µg/kg	100 µg/kg	2000 µg/kg	900 µg/kg	8000 µg/kg	50 µg/kg	250 µg/kg
Northern Europe	2.4	0.4	0.0	0.0	6.2	0.1	21.5	1.0	0.2	0.0
Central Europe	2.6	1.0	1.3	0.0	13.0	0.4	20.4	0.9	0.3	0.1
Southern Europe	7.4	2.1	3.3	0.0	11.8	0.2	11.7	0.5	0.9	0.2
Eastern Europe	5.4	0.2	0.3	0.0	4.8	0.1	4.3	0.1	0.4	0.2
North America	6.2	3.4	3.9	0.2	16.8	0.6	19.1	0.8	0.1	0.0
Central America	3.6	0.0	3.8	0.0	10.7	0.0	8.1	0.0	0.0	0.0
South America	6.5	1.3	8.4	0.2	13.1	0.2	5.1	0.0	0.8	0.5
Middle East/North Africa	7.5	3.5	1.1	0.0	8.6	0.0	5.6	0.0	0.9	0.0
Sub-Saharan Africa	59.1	38.5	1.0	0.0	5.0	0.0	7.0	0.0	4.2	0.8
South Africa	3.3	1.2	2.0	0.0	8.1	0.3	11.1	0.4	0.1	0.0
Oceania	3.0	1.0	0.6	0.0	11.1	0.7	5.1	1.1	0.1	0.0
South Asia	61.1	41.1	0.5	0.0	2.0	0.0	1.5	0.0	2.4	0.4
Southeast Asia	37.9	20.9	2.0	0.0	10.1	0.4	4.8	0.5	0.4	0.0
East Asia	10.2	6.6	3.9	0.0	27.3	1.3	20.6	0.7	0.3	0.0

^1^ Percentage of samples exceeding lowest or highest EU maximum levels or guidance values for mycotoxins in feed (including the lowest maximum level or guidance value stipulated for any commodity or consuming species and higher maximum levels or guidance values stipulated excluding limits for maize by-products and for oat husks). In case of fumonisins, the sum of fumonisins B_1_, B_2_, and B_3_ was compared to the EU guidance values, although the guidance values refer to the sum of fumonisins B_1_ and B_2_.

**Table 3:** Global co-occurrence of mycotoxins in finished feed, maize, and wheat.

Mycotoxin Combination^1^	Finished Feed	Maize	Wheat
AFB_1_ + DON	14%	15%	5%
AFB_1_ + ZEN	14%	11%	3%
AFB_1_ + FUM	22%	24%	1%
AFB_1_ + OTA	12%	2%	1%
AFB_1_ + T-2	3%	3%	5%
DON + ZEN	48%	39%	28%
DON + FUM	48%	49%	8%
DON + OTA	15%	3%	6%
DON + T-2	19%	10%	14%
ZEN + FUM	43%	37%	5%
ZEN + OTA	14%	2%	2%
ZEN + T-2	18%	9%	9%
FUM + OTA	17%	4%	1%
FUM + T-2	11%	9%	3%
OTA + T-2	7%	1%	2%

^1^ AFB_1_–aflatoxin B_1_; DON–deoxynivalenol; ZEN–zearalenone; FUM–fumonisins (sum of fumonisins B_1_, B_2_ and B_3_); OTA–ochratoxin A; T-2–T-2 toxin.

**Table 4:** Sample numbers per commodity and region.

	Finished Feed	Maize	Maize DDGS	Maize Silage	Soybean Grains	Soybean Meal	Wheat	Barley	Rice	Other Feed	Total
**Northern Europe**	236	20	5	43	6	6	378	555	0	709	**1958**
**Central Europe**	5328	3576	16	1431	208	67	3866	3172	27	3345	**21,036**
**Southern Europe**	1463	869	8	177	78	36	197	91	4	604	**3527**
**Eastern Europe**	1183	287	0	71	29	55	349	115	1	292	**2382**
**North America**	1082	1959	118	481	93	69	109	21	1	1538	**5471**
**Central America**	206	83	0	14	16	8	4	0	0	36	**367**
**South America**	3428	8407	0	59	362	2233	205	2	10	2626	**17,332**
**Middle East/North Africa**	543	178	4	46	38	13	69	11	0	173	**1075**
**Sub-Saharan Africa**	92	40	0	1	9	7	9	4	0	46	**208**
**South Africa**	324	306	0	111	32	7	12	5	1	279	**1077**
**Oceania**	222	35	14	262	11	26	260	128	4	733	**1695**
**South Asia**	557	211	1	5	43	38	17	0	22	242	**1136**
**Southeast Asia**	1826	895	73	0	170	163	151	2	87	943	**4310**
**East Asia**	5098	2930	150	1614	91	91	521	36	113	2588	**13,232**
**Central Asia**	0	2	0	0	0	0	13	0	0	0	**15**
**Total**	**21,588**	**19,798**	**389**	**4315**	**1186**	**2819**	**6160**	**4142**	**270**	**14,154**	**74,821**

**Table 5:** Mycotoxin analysis of feed samples.

Analyzer	Sample Number	Method^1^	Limits of Detection (µg/kg)^1^							
			AFB_1_	ZEN	DON	FB_1_	FB_2_	FB_3_	OTA	T-2
Romer Labs (Tulln, Austria)	13,438	ELISA	1	20	200	200	200	n.a.	1.9	10
Romer Labs (Tulln, Austria)	10,873	HPLC	0.2	4	20	20	20	n.a.	0.2	2
Romer Labs (Tulln, Austria)	9747	LC-MS/MS	0.2	4	20	20	20	n.a.	0.2	2
Romer Labs (Singapore)	7052	LC-MS/MS	0.5	10	10	10	10	n.a.	0.5	10
BIOMIN (Shanghai, China)	5282	HPLC	3	30	150	300	300	n.a.	1.7	n.a.
Romer Labs (Tulln, Austria) test strips operated by BIOMIN and commercial customers	4769	ELISA	3	20	200	200	200	n.a.	2	20
**Romer Labs (Union, USA)**	4689	HPLC	0.2	4	20	20	20	n.a.	0.2	2
**Romer Labs (Union, USA)**	3636	LC-MS/MS	0.2	4	20	20	20	n.a.	0.2	2
**Biofarma (Córdoba, Argentina)**	3058	HPLC	1	20	250	250	250	n.a.	n.a.	20
**IFA-Tulln^2^**	2696	LC-MS/MS	1.5	0.3	1.5	4	4	4	1.5	10
**Labocéa (Plouzané, France)**	1665	HPLC	0.2	2.8	1.2	10	10	n.a.	0.06	25
**SAMITEC (Santa Maria, Brazil)**	1191	HPLC	1	20	200	125	125	n.a.	2	100
**Romer Labs (Union, USA)**	999	ELISA	1	20	200	200	200	n.a.	1.9	10
**Spectrum^®^, VNITIP (Sergiev Posad, Russia)**	936	LC-MS/MS	2.01	1.8	7.2	5.4	5.4	n.a.	1.08	3.62
**Biofarma (Córdoba, Argentina)**	909	ELISA	1	20	200	125	125	n.a.	n.a.	100
**BIOMIN (Shanghai, China)**	760	ELISA	1	20	200	200	200	n.a.	1.9	10
**Bayrischer Tiergesund-heitsdienst (Poing, Germany)**	642	ELISA	n.a.	50	100	n.a.	n.a.	n.a.	n.a.	n.a.
**Royal Agricultural Stations (Thailand)**	616	HPLC	0.5	10	10	10	10	n.a.	0.5	10
**BIOMIN (Binh Duong, Vietnam)**	405	HPLC	1	10	10	25	25	n.a.	1	15
**ISU (Ames, Iowa)**	403	LC-MS/MS	5	100	100	100	100	n.a.	100	100
**BioCheck (Leipzig, Germany)**	290	ELISA	0.5	6	10	25	25	n.a.	0.2	3
**BioCheck (Leipzig, Germany)**	206	HPLC	2.7	0.5	3	1.5	1.5	n.a.	0.5	0.5
**Actlabs (Ancaster, Canada)**	190	LC-MS/MS	1	30	60	100	100	n.a.	3	60
**LAMIC (Santa Maria, Brazil)**	99	HPLC	1	20	200	125	125	n.a.	n.a.	100
**LUFA (Oldenburg, Germany)**	80	ELISA	n.a.	10	300	n.a.	n.a.	n.a.	n.a.	n.a.
**Uniwersytet Bydgoszcz (Bydgoszcz, Poland)**	41	HPLC	n.a.	0.2	6	5	5	n.a.	1.2	0.6
**Southern African Grain Laboratory (The Willows, South Africa)**	36	LC-MS/MS	5	50	100	20	20	20	5	10
**SGS (Hamburg, Germany)**	35	LC-MS/MS	n.a.	5	10	n.a.	n.a.	n.a.	n.a.	n.a.
**Tierklinik (St. Veit, Austria)**	27	ELISA	n.a.	10	200	n.a.	n.a.	n.a.	n.a.	n.a.
**SVÚ (Olomouc, Czech Republic)**	27	ELISA	n.a.	50	100	n.a.	n.a.	n.a.	n.a.	65
**SVÚ (Jihlava, Czech Republic)**	9	ELISA	n.a.	50	100	n.a.	n.a.	n.a.	n.a.	n.a.
**Sevaron Poradenství (Brno, Czech Republic)**	7	ELISA	n.a.	30	100	n.a.	n.a.	n.a.	n.a.	30
**University Latvia (Riga, Latvia)**	7	HPLC	1	150	200	n.a.	n.a.	n.a.	n.a.	100
**Zemědělská oblastní laboratoř (Chotýšany, Czech Republic)**	1	ELISA	n.a.	20	n.a.	n.a.	n.a.	n.a.	n.a.	n.a.

^1^ Abbreviations: AFB_1_–aflatoxin B_1_; DON–deoxynivalenol; ZEN–zearalenone; FB_1_–fumonisin B_1_; FB_2_–fumonisin B_2_; FB_3_–fumonisin B_3_; OTA–ochratoxin A; T-2–T-2 toxin; ELISA–enzyme linked immunosorbent assay; HPLC–high performance liquid chromatography; LC-MS/MS–liquid chromatography tandem mass spectrometry; n.a.–not analyzed. ^2^ Samples were analyzed at the Department of Agrobiotechnology (IFA-Tulln) at the University of Natural Resources and Life Sciences Vienna (BOKU) in Tulln, Austria as described by Kovalsky et al. [14].
